# Cross-Subject Emotion Recognition Brain–Computer Interface Based on fNIRS and DBJNet

**DOI:** 10.34133/cbsystems.0045

**Published:** 2023-07-27

**Authors:** Xiaopeng Si, Huang He, Jiayue Yu, Dong Ming

**Affiliations:** ^1^ Academy of Medical Engineering and Translational Medicine, Tianjin University, Tianjin 300072, People’s Republic of China.; ^2^ Tianjin Key Laboratory of Brain Science and Neural Engineering, Tianjin University, Tianjin 300072, People’s Republic of China.; ^3^ Tianjin International Engineering Institute, Tianjin University, Tianjin 300072, People’s Republic of China.

## Abstract

Functional near-infrared spectroscopy (fNIRS) is a noninvasive brain imaging technique that has gradually been applied in emotion recognition research due to its advantages of high spatial resolution, real time, and convenience. However, the current research on emotion recognition based on fNIRS is mainly limited to within-subject, and there is a lack of related work on emotion recognition across subjects. Therefore, in this paper, we designed an emotion evoking experiment with videos as stimuli and constructed the fNIRS emotion recognition database. On this basis, deep learning technology was introduced for the first time, and a dual-branch joint network (DBJNet) was constructed, creating the ability to generalize the model to new participants. The decoding performance obtained by the proposed model shows that fNIRS can effectively distinguish positive versus neutral versus negative emotions (accuracy is 74.8%, F1 score is 72.9%), and the decoding performance on the 2-category emotion recognition task of distinguishing positive versus neutral (accuracy is 89.5%, F1 score is 88.3%), negative versus neutral (accuracy is 91.7%, F1 score is 91.1%) proved fNIRS has a powerful ability to decode emotions. Furthermore, the results of the ablation study of the model structure demonstrate that the joint convolutional neural network branch and the statistical branch achieve the highest decoding performance. The work in this paper is expected to facilitate the development of fNIRS affective brain–computer interface.

## Introduction

Emotion recognition is an important part of human–computer interaction, and it is also the first step to understanding subject's feelings [1]. It has broad application prospects, especially in the diagnosis and treatment of mental diseases [2], and is one of the important research topics in the field of brain–computer interaction [3,4]. Traditional emotion recognition methods are mainly based on information such as questionnaires, facial expressions [5,6], and voice [7]. However, these methods face problems such as strong subjectivity and susceptibility to interference. Currently, brain imaging techniques allow researchers to noninvasively detect neural activity signals during emotion evoking [8,9], allowing the identification of emotional categories by neural activity signatures. Compared with behavioral characteristics (such as facial expressions, human voice, etc.), the neural activity related to emotion is difficult to hide [10]. In addition, compared with physiological signals (skin electricity, myoelectricity, electrocardiogram, etc.), neural activity has a higher specificity for different emotional categories and contains more effective feature information that can distinguish emotions [11].

Functional near-infrared spectroscopy (fNIRS) is a noninvasive neuroimaging technique that noninvasively measures changes in blood oxygen levels in the cerebral cortex. Compared to functional magnetic resonance imaging, fNIRS does not require participants to lie in a confined space and allows for brain imaging in a more natural state, making it applicable to a wider range of participants [12]. Compared to electroencephalography (EEG), fNIRS has higher spatial resolution [13] and is less susceptible to motion artifacts [14]. In conclusion, fNIRS, as a portable and cost-effective functional neuroimaging modality, together with its relatively high spatial resolution and practical sensory setup, is particularly suitable for studying brain activity related to cognitive and emotional processing [15–17].

In recent years, researchers have carried out a series of studies on emotion recognition based on fNIRS technology. They conducted different emotion-evoking experiments on the subjects, collected the fNIRS signals of their cerebral cortex, and used machine learning and other methods to identify emotions. Among them, the researchers used fNIRS to perform intrasubject emotion recognition on the emotions caused by pictures [18], audio [19], and video [20], revealing the feasibility of decoding emotional states based on fNIRS. Furthermore, Heger et al. [20] estimates a short time window of 5 s to continuously recognize emotional states, and its valence-based binary recognition performance can reach 61.2%. Hu et al. [21] reports for the first time identifiable discrete positive emotions using fNIRS signals, providing support for the realization of more fine-grained emotion recognition systems with subdivided positive emotion categories.

Although the research on emotion decoding based on fNIRS has made some progress, there are still the following deficiencies. First, cross-subject emotion recognition is of great significance for improving the effectiveness, reliability, and universality of emotion recognition technology. However, current fNIRS emotion recognition research is limited to within-subject emotion recognition, and to our knowledge, the study in [22] is the only fNIRS-based cross-subject emotion recognition work. Second, the current classification algorithms used in the field of fNIRS emotion recognition are all based on traditional machine learning models, without introducing deep learning methods. Notably, the application of deep learning techniques to fNIRS research has been shown to alleviate many of the obstacles in fNIRS research, such as lengthy data preprocessing or small sample sizes, while enabling comparable or improved classification accuracy [23].

To solve the above problems, in this paper, we construct a cross-subject emotion recognition framework based on fNIRS. The main contributions of this article are as follows:

1. The fNIRS emotion recognition database was constructed, and the database contained the data of 18 subjects.
2. In the field of fNIRS emotion recognition, deep learning technology was introduced for the first time, and the performance of emotion recognition was greatly improved by constructing fNIRS cross-subject emotion recognition network, dual-branch joint network (DBJNet), and extensive ablation research and comparative experiments were carried out to prove our model superiority.
3. For the first time, the decoding research of positive versus neutral versus negative emotions was carried out based on fNIRS signals, and it was found that fNIRS has a strong ability to identify positive versus neutral emotions, negative versus neutral emotions.

The remainder of the article is organized as follows: Materials and Methods presents the materials and methods of this study. Results presents the results. Discussion presents the discussion of this study.

## Materials and Methods

### Subjects

In this experiment, a total of 18 healthy subjects (mean ± SD age = 23.4 ± 0.8 years, 12 males and 6 females) were recruited to participate in the video emotional-evoking experiment (Table 1). The subjects were all right-handed healthy college students whose mother tongue was Chinese and had no auditory or cognitive impairment and no history of neurological diseases. Before the experiment, the subjects were required to ensure adequate sleep and not to drink alcohol, coffee, and any other substances that might affect the experiment. The experiment passed the ethical review, and all subjects were informed of the complete process of the experiment and obtained informed consent before the experiment. All subjects completed all experiments at one time.

**Table 1.** Subject information.

Subject	Gender	Age (years)	Handedness
S1	Male	24	Right hand
S2	Male	23	Right hand
S3	Male	25	Right hand
S4	Male	22	Right hand
S5	Male	24	Right hand
S6	Male	23	Right hand
S7	Male	23	Right hand
S8	Male	23	Right hand
S9	Male	24	Right hand
S10	Female	23	Right hand
S11	Male	23	Right hand
S12	Female	23	Right hand
S13	Female	24	Right hand
S14	Female	23	Right hand
S15	Female	23	Right hand
S16	Female	23	Right hand
S17	Male	24	Right hand
S18	Male	24	Right hand

### Equipment setup

**Fig. 1.**
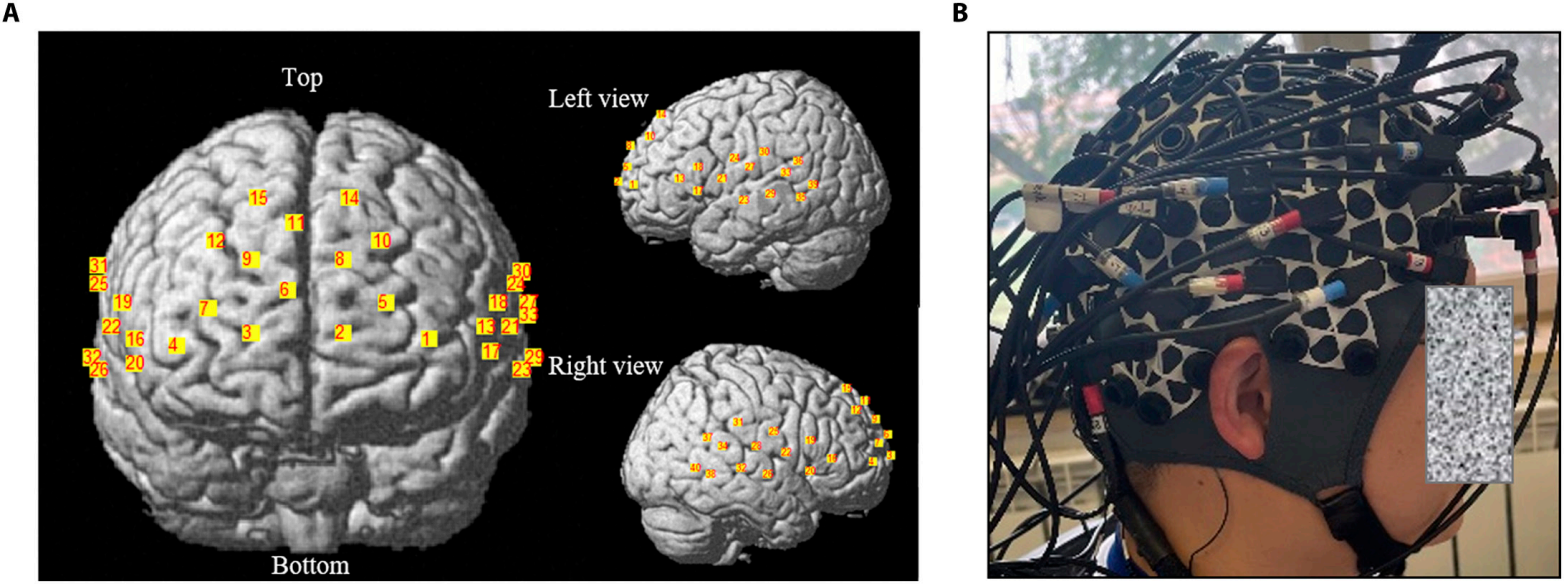
fNIRS channel configuration information. (A) Topographic distribution of fNIRS recording channels. (B) Participant wearing fNIRS sensors.

Experiments are performed in a controlled laboratory environment. In this study, fNIRS manufactured by Shimadzu (LABNIRS, Shimadzu Corp., Kyoto, Japan) was used to collect hemodynamic signals, the sampling rate was set to 4 Hz, and the light wavelengths were 780, 805, and 830 nm. By using 40 channels composed of 15 light sources and 16 detectors, the bilateral middle frontal gyrus, inferior frontal gyrus, middle temporal gyrus, superior temporal gyrus, precentral gyrus, postcentral gyrus, and superior frontal gyrus were covered (Fig. 1).

### Stimulus material

The experiment used video stimulation to induce emotions and selected 3 types of emotions: positive emotions (happy), neutral emotions, and negative emotions (sadness) as the stimulating emotions. To ensure the effectiveness of the video stimulus materials, some of the video stimuli are selected from the SEED-IV [24] dataset, and some of them are high-scoring movies selected according to Douban scores. Afterward, the stimulus materials were screened twice to obtain video materials with better stimulation effects. The stimulus screening process is as follows:

1. According to the SEED-IV dataset and Douban score, 12 happy stimuli, 12 sad stimuli, and 24 neutral stimuli were selected. Among them, Douban rating is a system where Douban users rate cultural products such as movies, TV dramas, music, etc., with a rating range of 1 to 10 points. We chose to use the Douban score to screen for stimuli because Douban score is a relatively objective evaluation indicator that can reflect the overall evaluation of a cultural product by the public.

2. Eight healthy college students with normal hearing were recruited as raters, and they scored the video material from the 2 dimensions of emotional valence and arousal. The scores were scored using the self-assessment manikin scale [25], with a score range of 1 to 5.

3. According to the valence, the 6 happiest videos, 6 saddest videos, and 12 videos with valences closest to 3 are selected. When the valence was the same, emotional videos with higher arousal (happy and sad) and neutral videos with arousal closer to 3 were selected according to the arousal. When the degree of arousal is the same, emotional videos with higher dominance (happy and sad) and neutral videos with a dominance closer to 3 are selected according to the dominance. It is worth noting that selecting neutral videos with emotional arousal or dominance scores close to 3 as experimental stimuli can maintain relative consistency of visual stimuli for participants, while avoiding overstimulation, thus allowing participants to exhibit a neutral emotional state as much as possible while watching such videos.

4. After the objective and reasonable screening, 6 happy videos, 6 sad videos, and 12 neutral videos were obtained as emotional stimulation materials.

### Protocol

**Fig. 2.**
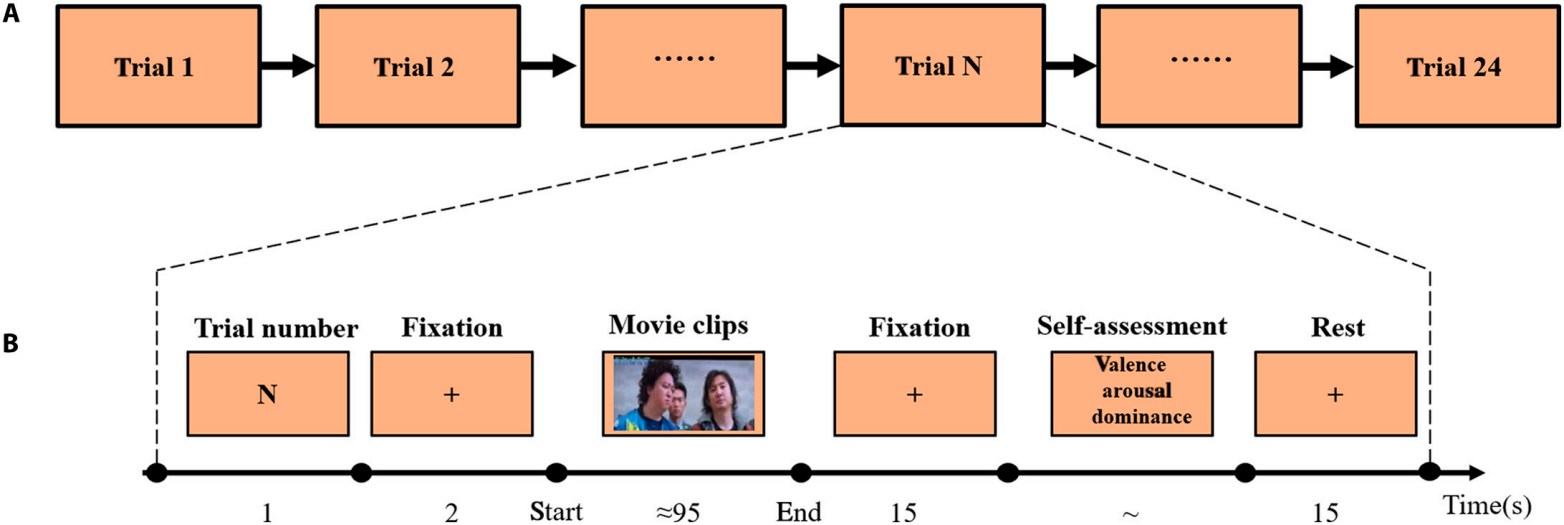
A fNIRS experimental paradigm for video emotion evoking. (A) Process relationship between trials. (B) The sequence of processes within a trial.

The experimental paradigm is shown in Fig. 2. The whole experiment is divided into 2 task blocks (block), and each block contains 12 trials. Each trial first displays the trial sequence number for 1 s, followed by 2 s of concentration, and then the subject starts to watch the video and then rests for 15 s after watching the video. The design of this duration is set according to the characteristics of fNIRS data and can meet the time required for the blood oxygen signal to return to the baseline state. After watching the video, the subjects rated the video as a whole according to their emotional state. The order of scoring was first to evaluate the valence, then to evaluate the degree of arousal, and finally to evaluate the degree of dominance. After the evaluation, the subjects were given 15 s [22] to rest and concentrate, as the event-triggered hemodynamic response usually shows an increase in signal lasting 10 to 12 s to peak and return to baseline [26], this time can help subjects calm down the video and evaluation impact. We require the participants to remain still during the 15-s rest period. A cross is displayed on the screen during this time to attract their attention, and the information displayed on the screen is consistent with the viewing instructions shown 2 s prior to the start of the video. The entire experiment was performed for about 70 min in total. It is worth noting that for the video playback sequence, we will intersperse a neutral emotion video between the positive and negative emotion videos, using neutral emotion movie clips as a buffer between the 2 opposite emotions to avoid sudden changes in emotions [27].

### System framework

**Fig. 3.**
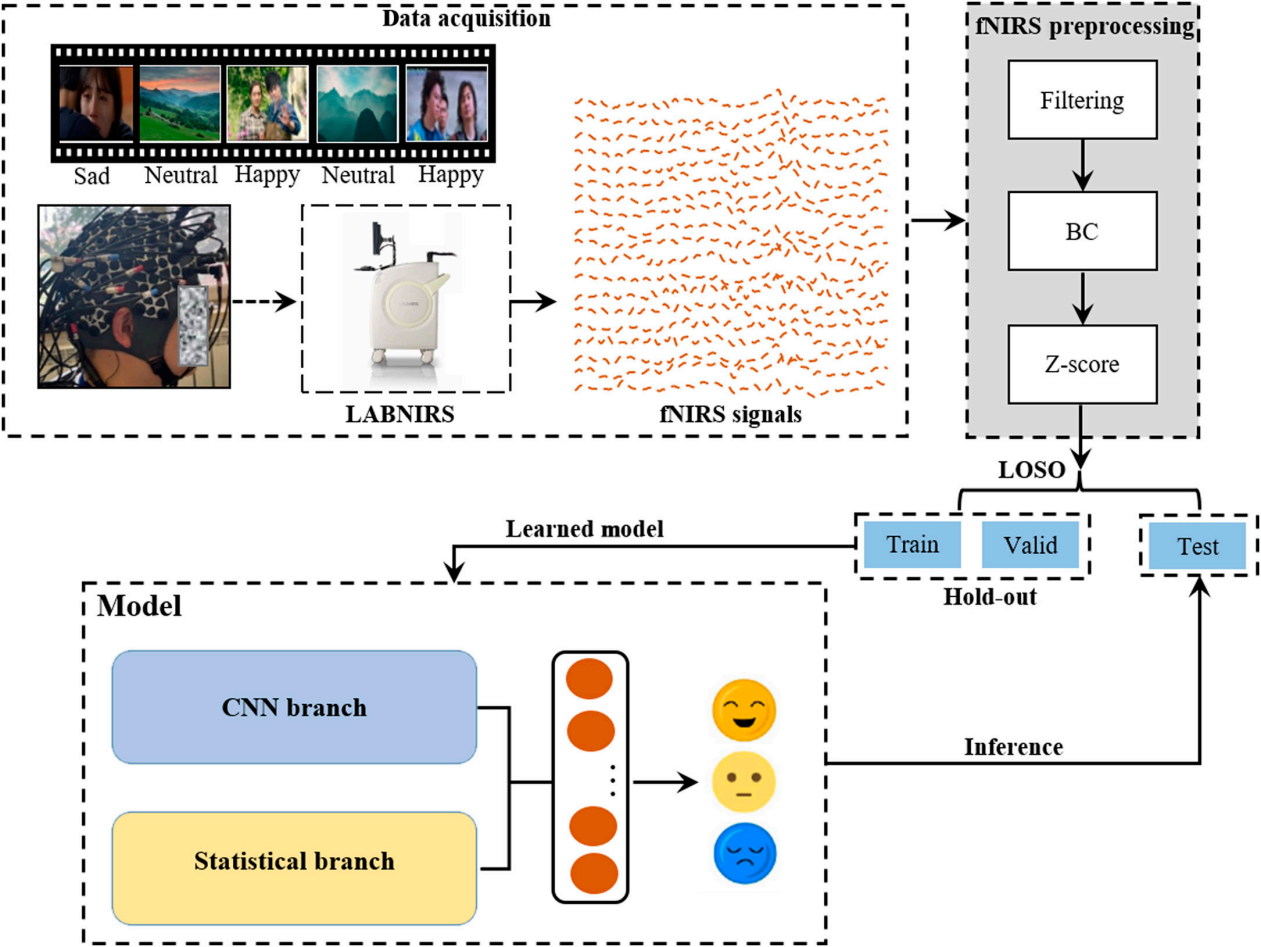
Our proposed system architecture for fNIRS-based cross-subject emotion recognition. BC, baseline correction; LOSO, leave-one-subject-out cross-validation. Hold-out: 80% is used as the training set, and 20% is used as the verification set.

Figure 3 shows our proposed framework for fNIRS-based emotion recognition. First, participants collected fNIRS signal data while viewing emotional movie clips. Second, the raw fNIRS signal is preprocessed. Then, the data were divided into the training set, verification set, and test set, where the training and verification data are input into the constructed deep learning model to train the emotion classification model. Finally, the performance of the trained emotion classification model was evaluated on the test data.

### Data acquisition

Before data collection, the optical path settings of each fNIRS channel were adjusted for the subjects, trying to make the signal quality of each channel meet the good standard required by the equipment. During data collection, subjects sat in a comfortable chair and watched a screen. The device will record the blood oxygen concentration (oxygenated Hb [HbO] and deoxygenated Hb [HbR]) of the subject during the whole experiment. Labels 1, 2, and 3 were recorded to distinguish trials of different emotion categories while the subjects were watching negative, neutral, and positive emotion movie clips, and these data were used as subsequent classification labels.

### fNIRS signal to preprocess

First, we used a third-order 0.01- to 0.5-Hz band-pass IIR Butterworth filter to remove instrumental and physiological noise interference. Second, HbO data were extracted for each trial, and baseline correction was performed by subtracting the mean response for the 5-s duration before the stimulus. Related studies have shown that hemodynamic responses triggered by events typically return to baseline within 10 to 12 s [26]. Therefore, in this study, the 5 s before the start of the video were chosen as the baseline, excluding 1 s for viewing the trial number and 2 s for viewing the instructions. We used the last 2 s of the 15-s rest period, which allowed for a 13-s period to recover from any potential movement effects during the previous self-evaluation phase of the trial. This amount of time was sufficient for the signal to return to baseline. Additionally, Moghimi et al. [19] has proved that the sentiment classification effect of HbO data is better than that of deoxygenated Hb data. The data for each channel were then normalized using Z-score. Finally, the signal corresponding to the last 40 s of each trial was intercepted to obtain the maximum emotional response [21].

### Model

**Fig. 4.**
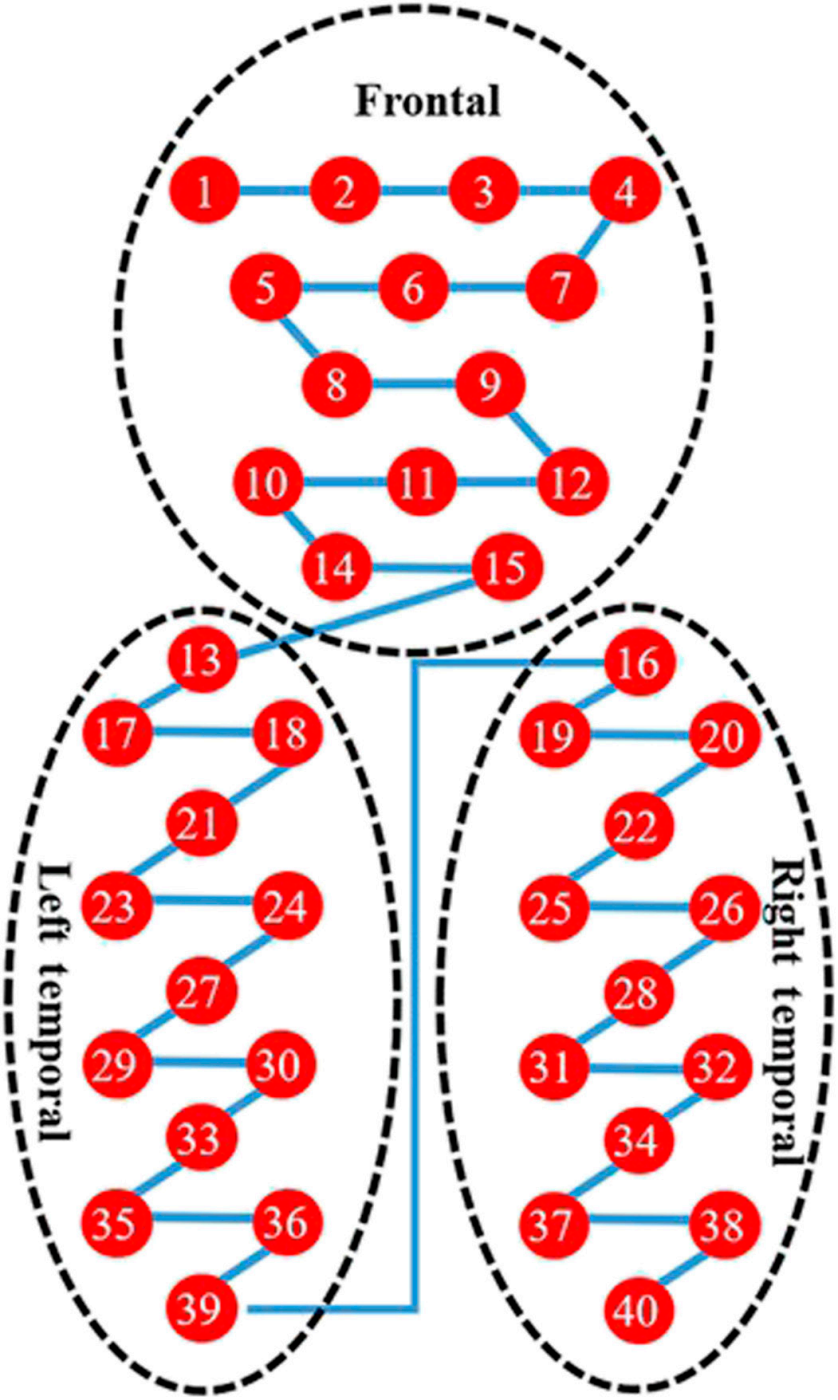
Dual-branch joint network (DBJNet) Architecture.

**Fig. 5.**
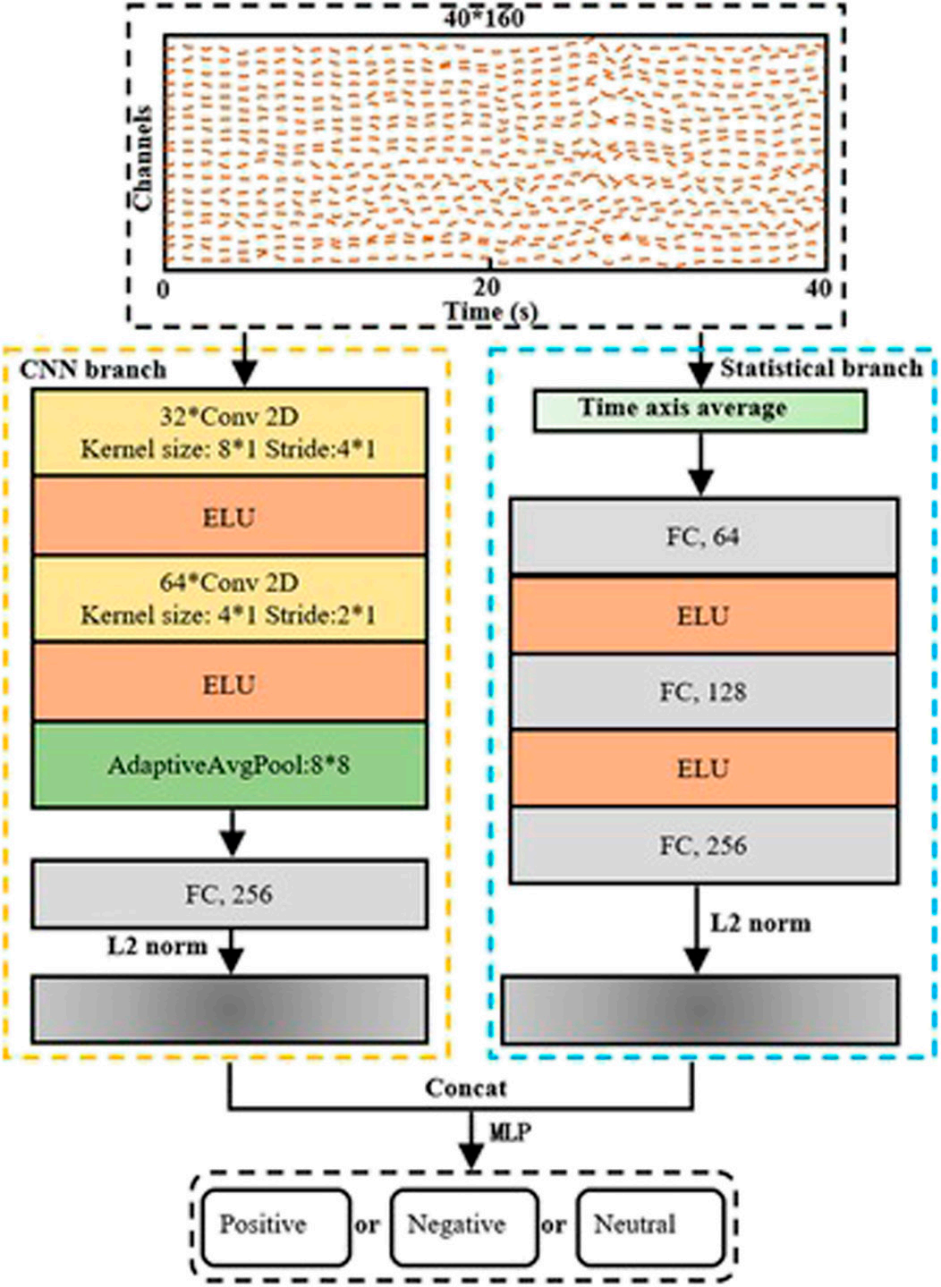
Schematic diagram of channel reordering. Sort spatially adjacent channels together. Among them, the red solid circles indicate the channels, and the blue solid lines indicate the order of the rearranged channels.

In this section, we specify the proposed model framework, called DBJNet, as shown in Fig. 4, to combine the signal characteristics of high spatial resolution of fNIRS, DBJNet is divided into 2 parts, namely, (a) convolutional neural network (CNN) branch, which extracts local spatial features based on spatial convolution and (b) Statistical branch, which extracts the global time average response feature of each channel. Below, this part will be discussed in detail. It is worth noting that in order to guarantee the spatial connection between channels, we reorder the channels in the order of adjacent channels, i.e., put adjacent channels together. The sequence before and after channel rearrangement is shown in Fig. 5. The code for the model has been published on GitHub (https://github.com/ThreePoundUniverse/2023CBS).

**Fig. 6.**
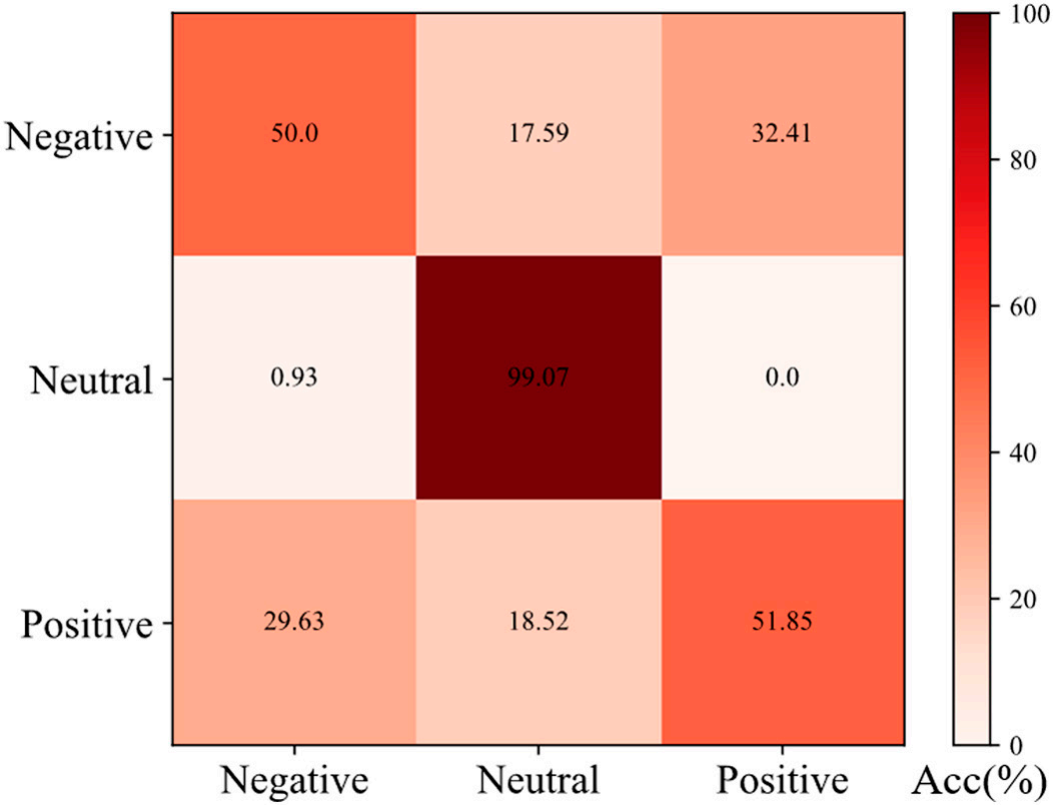
Three-category confusion matrix plot for positive, neutral, and negative emotions.

#### CNN branch

Due to the high spatial resolution of fNIRS and its more comprehensive spatial feature information compared to temporal feature information, we used 2-layer spatial convolution to fully extract local spatial features when designing CNN branches. Among them, the first layer of spatial convolution initially extracts spatial features, the number of convolution kernels is 32, the kernel size is (8, 1), and the stride is (4, 1), and then the exponential linear units (ELU) is used to activate layer [28], which performs a nonlinear transformation on the signal. The second layer of spatial convolution further extracts spatial features. The number of convolution kernels is 64, the kernel size is (4, 1), and the stride is (2, 1), followed by an ELU activation layer. Then, use the adaptive average pooling layer to reduce the time and space dimensions of the signal to 8. This step compresses the time dimension and extracts the local time-scale average response features. Finally, after flattening the dimensions of the output signal, a fully connected layer is used to transfer and transform the abstract representation captured by the CNN branch and compress the dimension of the representation vector to 256 dimensions.

#### Statistical branch

Statistical features commonly used in brain–computer interface (BCI) fNIRS research include signal mean, variance, kurtosis, skewness, peak, and slope, among which signal mean is widely used in the field of fNIRS emotion recognition [18,21] and proved to be the most effective statistical feature. Therefore, we extracted the average response of the fNIRS signal as the feature input of the statistical branch of the model, and input the feature vector into 3 fully connected layers for feature extraction, and each fully connected layer was followed by an ELU activation layer. After multiple linear and nonlinear transformations of the fully connected layer and the activation layer, the high-dimensional features of the input data can be extracted, to better express the structure and information of the data, and finally expand the dimension of the representation vector to 256 dimensions, which is consistent with the CNN branch Corresponding.

The 2 characterization vectors of the CNN branch and the Statistical branch are respectively L2 normalized so that they are in the same data domain. Then, the two were concated and input to the fully connected layer for classification. Overall, the 2 branches together constitute the proposed model architecture, DBJNet. Among them, the CNN branch provides abstract spatial feature information and local time average response features, while the Statistical branch provides abstract global time average response features. Table 2 shows the specific parameter settings of DBJNet.

**Table 2.** The specific parameter setting of DBJNet.

Model	Step no.	Step name	Parameters	Output shape
CNN branch	1	Input	Input Shape=(40,160)	(1,40,160)
	2	Conv2D	Size=(8,1), Num=32, Elu, Stride=(4,1)	(32,9,160)
	3	Conv2D	Size=(4,1), Num=64, Elu, Stride=(2,1)	(64,3,160)
	4	AdaptiveAvgPool2D	Output_size=(4,4)	(64,8,8)
	5	Flatten & L2 norm	\	(64*8*8)
	6	FC	Linear units=256	256
Statistical branch	1	Input	Input Shape=(40,160)	(40,160)
	2	Mean	Dim=1	(40)
	3	FC	Linear units=64, ELU	(64)
	4	FC	Linear units=128, ELU	(128)
	5	FC	Linear units=256, ELU	(256)
	6	L2 norm	\	(256)
Combine	7	Concat	\	(512)
	8	FC	Linear units=3	3

### Implement details

The experiment uses the Adam optimizer, the initial learning rate is set to 1e-4, and the learning rate attenuation strategy is ReduceOnPlateau, that is, when the verification set loss does not decrease within 14 epochs, the learning rate is attenuated by 10%, and the batch size is set to 32. The number of epochs is 200, and the early stopping strategy is adopted.

### Performance evaluation

For the entire dataset, we employ leave-one-subject-out cross-validation. Specifically, one of the 18 subjects is used as the test set, 80% of the remaining data is used as the training set, and 20% is used as the verification set. During the training process, the parameter model with the highest accuracy of the verification set is saved and evaluated on the test set. The above process was repeated 18 times to ensure that each subject serves as a test set, and take the average of the test set accuracy of all folds as the final classification performance of the model.

The evaluation indicators of model performance are accuracy (ACC) and F1 score (F1).

## Results

In this section, we report the results of the proposed method in terms of accuracy and F1 score and compare them with classic and cutting-edge algorithms. In addition, we conducted ablation studies to reveal the role of each component in DBJNet.

### Three-category emotion recognition performance

We compared the proposed method with other deep learning models in terms of accuracy and F1 score, including EEGNet [28], CNN + NLSTM [29], and Tsception [30]. Table 3 shows the leave-one-out-subject cross-validation results for all methods on our fNIRS dataset. Our proposed method outperforms these models by about 16% on average on the classification level of positive versus neutral versus negative emotions. In addition, compared with support vector machines (SVMs) and linear discriminant analysis (LDA), the classification level of DBJNet on positive versus neutral versus negative 3 kinds of emotions is improved by about 14%.

**Table 3.** Comparison of the accuracy (ACC) and F1 scores (mean ± SD) of DBJNet and other methods on the fNIRS cross-subject emotion recognition task. Among them, Neg stands for Negative, Pos stands for Positive, and Neu stands for Neutral.

Model	Neg vs Pos		Neg vs Neu		Pos vs Neu		Pos vs Neu vs Neg	
	ACC (%)	F1 (%)	ACC (%)	F1 (%)	ACC (%)	F1 (%)	ACC (%)	F1 (%)
LDA	46.3 ± 16.5	45.1 ± 16.7	83.3 ± 9.4	81.9 ± 10.7	83.3 ± 7.9	82.0 ± 8.8	59.7 ± 7.4	57.3 ± 7.8
SVM	53.2 ± 13.4	51.3 ± 14.5	83.3 ± 7.6	81.7 ± 8.9	84.6 ± 7.5	83.7 ± 8.1	62.3 ± 6.3	59.1 ± 7.3
EEGNet[28]	51.9 ± 13.5	48.5 ± 15.2	75.9 ± 12.4	74.9 ± 12.4	73.8 ± 12.9	72.3 ± 13.5	60.9 ± 9.6	56.0 ± 10.2
CNN + NLSTM[29]	52.3 ± 13.3	50.1 ± 12.8	78.4 ± 11.7	77.6 ± 11.6	74.1 ± 12.7	73.5 ± 12.8	57.6 ± 8.9	54.8 ± 9.8
Tsception[30]	55.1 ± 11.8	46.7 ± 16.5	74.7 ± 10.3	73.8 ± 10.3	74.7 ± 9.5	72.3 ± 11.5	59.2 ± 8.0	57.1 ± 8.6
DBJNet	61.1 ± 11.8	59.6 ± 12.0	91.7 ± 5.6	91.1 ± 6.3	89.5 ± 7.8	88.3 ± 9.2	74.8 ± 9.4	72.9 ± 10.5

The classification performance of the traditional machine learning algorithms in Table 3 is similar to the 3 compared deep learning models, and the machine learning model is slightly higher than the compared deep learning models. We speculate that this is due to the relatively large modal gap between EEG and fNIRS, and the model that is suitable for EEG signals is not necessarily suitable for fNIRS signals. It is worth noting that the recognition performance of our proposed model on all 3 emotions is much higher than that of the compared methods, which demonstrates the superiority of our DBJNet on the fNIRS emotion recognition task.

It is worth noting that the results in Table 3 show that our 3-class classification accuracy is higher than the binary classification of positive versus negative. In response to this result, this paper draws a 3-category confusion matrix diagram (Fig. 6). We found that in the 3-category task, because the model is very effective in recognizing neutral emotions, the overall classification accuracy of the 3-category task is increased.

In summary, according to extensive comparisons with various methods, the proposed method shows superiority in the fNIRS cross-subject emotion recognition task, especially since its ability to distinguish positive versus neutral and negative versus neutral is very strong.

### Binary classification emotion recognition performance

The comparison of binary classification emotion recognition performance in Table 3 also demonstrates the superiority of the proposed method. Outperforms state-of-the-art deep learning models by an average of 8% on positive versus negative, by 15% on average on negative versus neutral, and by an average of 15% on positive versus neutral. In addition, compared with SVM and LDA, DBJNet improved by about 11%, 8%, and 6% on negative versus positive, negative versus neutral, and positive versus neutral, respectively. It is worth noting that DBJNet achieves a performance of about 90% on positive versus neutral and negative versus neutral, indicating that our method has a strong decoding ability in distinguishing positive versus neutral emotions, as well as negative versus neutral emotion tasks. Furthermore, we found that the decoding performance of positive versus neutral emotions, as well as negative versus neutral emotions, was much higher than that of positive versus negative emotions in all methods. This indicates that fNIRS has strong potential in distinguishing positive versus neutral emotions, as well as negative versus neutral emotions, and its decoding ability in positive from negative emotions needs further improvement.

### Dual-branch joint for maximum performance

To investigate how our model achieves such performance, we conduct ablation experiments on the proposed model. Table 4 examines the contribution of the CNN branch and the Statistical branch to the model performance. The experimental results indicate that, without the CNN branch, the ACC and F1 scores for negative versus positive emotion decreased by 2.3% and 2.1%, respectively. The ACC and F1 scores for negative versus neutral emotion decreased by 12.1% and 12.6%, respectively. The ACC and F1 scores for positive versus neutral emotion decreased by 13.6% and 13.7%, respectively. The ACC and F1 scores for negative versus neutral versus positive emotion decreased by 14.2% and 16.1%, respectively. Without the Statistical branch, the ACC and F1 scores for negative versus positive emotion decreased by 0.9% and 1.7%, respectively. The ACC and F1 scores for negative versus neutral emotion decreased by 5.3% and 6.3%, respectively. The ACC and F1 scores for positive versus neutral emotion decreased by 7.1% and 9.3%, respectively. The ACC and F1 scores for negative versus neutral versus positive emotion decreased by 11.4% and 16.3%, respectively. Comparatively, the contribution of the Statistical branch is superior to the CNN branch, but the joint use of both branches can achieve the highest decoding performance, therefore, the 2 are indispensable to each other.

**Table 4.** Results of ablation experiments on 2 branch components of DBJNet. Among them, Neg stands for Negative, Pos stands for Positive, and Neu stands for Neutral.

CNN branch	Statistical branch	Neg vs Pos		Neg vs Neu		Pos vs Neu		Pos vs Neu vs Neg	
		ACC (%)	F 1(%)	ACC (%)	F1 (%)	ACC (%)	F1 (%)	ACC (%)	F1 (%)
✔	✘	58.8 ± 11.6	56.5 ± 12.5	79.6 ± 10.3	78.5 ± 10.6	75.9 ± 11.1	74.6 ± 11.0	60.6 ± 10.9	56.8 ± 11.7
✘	✔	60.2 ± 12.6	57.9 ± 13.9	86.4 ± 6.7	84.8 ± 8.1	82.4 ± 8.3	79.0 ± 11.6	63.4 ± 5.7	56.6 ± 78.2
✔	✔	61.1 ± 11.8	59.6 ± 12.0	91.7 ± 5.6	91.1 ± 6.3	89.5 ± 7.8	88.3 ± 9.2	74.8 ± 9.4	72.9 ± 10.5

## Discussion

In this paper, we established the video emotional stimulus paradigm and constructed the fNIRS emotion recognition database. In the field of fNIRS emotion recognition, deep learning technology is introduced for the first time, and the constructed DBJNet achieves the best decoding performance among all compared methods, proving the superiority of the proposed method. In addition, the model's ablation study results show that the joint CNN branch and the Statistical branch can achieve the highest decoding performance, which shows the rationality of our model structure design. Notably, all methods performed better on the task of distinguishing negative versus neutral emotions, and positive versus neutral emotions. In particular, our method achieves 90% emotion decoding performance, which demonstrates the strong capability of fNIRS-based emotion recognition in decoding positive versus neutral, and negative versus neutral.

For the first time, we realized the decoding research of positive versus neutral versus negative emotions in the field of fNIRS emotion recognition and introduced the deep learning model, which greatly improved the performance of emotion decoding. Furthermore, we set up an ablation study of the model structure, demonstrating the rationality and superiority of the proposed model. Therefore, our work greatly promotes the development of the fNIRS emotion recognition field and demonstrates the great potential of fNIRS for emotion recognition tasks.

Compared with related research [22], we strictly divide the data into the training set, verification set, and test set, and the division of datasets, which is more scientific and reasonable. In addition, we outperform [22] by about 25% on the task of decoding negative versus neutral and positive versus neutral emotions, far surpassing state-of-the-art performance.

However, this study still has some limitations. First of all, our positive emotions are only happy, our negative emotions are only sad, and the subdivision of emotions is limited. In addition, our results show that the constructed fNIRS cross-subject emotion recognition framework has poor decoding performance for positive versus negative emotions, with an accuracy of 61%. This aspect needs to be further improved. We speculate that the reason for limiting the ability of fNIRS to distinguish between positive and negative emotions may be due to the problem of brain area coverage, that is, the specific processing brain areas of positive and negative emotions are not taken into account when designing fNIRS channel layout. Third, we use the trial as the data unit [22] instead of sliding window slices [20], which cannot meet the requirements of continuous emotion recognition in actual application scenarios.

In the future, we will address the above limitations. We consider introducing more subdivided emotion categories to enrich the types of emotion recognition, for example, fear, disgust, etc. in negative emotions, and amusement, inspiration, etc. in positive emotions. In addition, the performance of both positive and negative emotion decoding is further improved by means of data augmentation [31] or adding fNIRS channel layout. Further, continuous emotion recognition based on fNIRS is studied by using sliding window slices [20]. Overall, existing and future research can promote the application and development of fNIRS emotion recognition BCI.

## Data Availability

The data used to support the findings of this study are available from the corresponding author upon request.
